# Older Adults Show Altered Network Connectivity during Fairness Decisions with Similar and Dissimilar Partners

**DOI:** 10.1101/2025.08.13.670194

**Published:** 2025-08-18

**Authors:** Yi Yang, Rita M. Ludwig, James B. Wyngaarden, Derrick Dwamena, Katherine Hackett, Tania Giovannetti, Dominic S. Fareri, David V. Smith

**Affiliations:** (1) Temple University, Philadelphia, USA; (2) University of Pennsylvania, Philadelphia, USA; (3) Icahn School of Medicine at Mount Sinai, New York, USA; (4) Adelphi University, Garden City, USA

**Keywords:** Decision making, Economic game, Social context, Fairness, Functional network connectivity

## Abstract

**Objectives:**

The ability to navigate diverse social contexts, such as interacting with different individuals, is crucial across the lifespan and has implications for fraud susceptibility. However, the neural mechanisms supporting social decisions in older adults within varied social contexts remain largely unknown. This study investigated how age and partner similarity modulate neural activation and network connectivity during fairness-related decision making and whether individual differences in behavioral sensitivity to fairness norm violations correlate with neural activities.

**Methods:**

Younger (18–35) and older (65–80) adults underwent fMRI while playing an ultimatum game with ostensible partners of a similar or dissimilar age. We used regression to model choice behavior and whole-brain fMRI analyses to examine functional activation and connectivity of the Default Mode (DMN) and Executive Control (ECN) networks.

**Results:**

Behaviorally, choices did not differ by age, partner similarity, or their interactions. In contrast, neuroimaging revealed interactions between age, partner similarity, and individual sensitivity to norm violation. Younger adults exhibited positive DMN-anterior cingulate connectivity when interacting with a similar-aged partner, whereas older adults showed a negative connectivity. Furthermore, younger adults demonstrated a negative correlation between their sensitivity to norm violation and the partner-identity effect in ECN-medial prefrontal cortex connectivity, whereas older adults showed a positive correlation.

**Discussion:**

Our findings indicate a brain-behavior dissociation where different neural mechanisms support similar behavioral outcomes across age groups. These opposing patterns of neural responses suggest age-related functional reorganizations, which may represent compensatory strategies that enable older adults to preserve behaviors similar to that of their younger counterparts.

## Introduction

Recent reports show that rates of financial insecurity and fraud are increasing among older Americans (Collins & Casey, 2017). Empirically-based policy solutions to address these worrying trends are necessary especially as the proportion of Americans entering advanced age continues to grow. Insights from neuroscientific research of aging-related brain changes during information processing, learning, and decision - making may help develop better protections for older people against scams and fraud. The present research adds to a growing body of literature specifically focused on financial decision-making in older adults by using a multi-trial ultimatum game with varying social context conditions related to age group similarity between participants and their game partners. The goal of this work is to illuminate associated neural structures and networks implicated during fairness-related choice responses within this game and identify any distinctions across adult age groups in the effects of partner similarity and individual sensitivity to norm violations.

Among the most common types of scams targeted at seniors are those that attempt to exploit social identity and relationships – a scammer may pretend to be a romantic interest, or to be calling on behalf of a relative in distress. Impersonation scams make up all top 5 scams that target older adults, causing a loss of 8 billion per year in older victims (Johnston, 2025). Given the apparent real-world influence of social context on financial decisions in older adults, we chose an ultimatum game with multiple social conditions (e.g., game partner identity) to study such decision -making in the lab. Ultimatum games are a take-it-or-leave-it class of economic games wherein a proposer divides an endowment with a responder, who then either chooses to accept or reject the offered split. If the responder rejects the offer, both parties walk away with nothing (Güth et al., 1982). These games are thought to elicit processes of social cognition, reward valuation, and learning by provoking a motivational conflict between economic self - interest and higher-order social goals of maintaining norms of fairness and reciprocity (Andreoni & Bernheim, 2009). Indeed, despite the economically rational choice being to always accept non-zero offers to increase personal net gain, responders frequently make the pyrrhic choice to reject unfair offers (Güth & Kocher, 2014).

The ultimatum game provides a powerful tool for studying fairness-related decision making. By manipulating the social context, such as age similarity between a player and their game partner, researchers can investigate how these social contextual factors influence the player’s choice to accept or reject an offer and related brain activations, and whether these behavioral and neural patterns differ between younger and older adults. At the behavioral level, research on age differences in the ultimatum game has yielded conflicting results; older adults have been reported to both accept more unfair offers than younger adults (Fernandes et al., 2019; Girardi et al., 2018) and, conversely, to reject them more frequently (Harlé & Sanfey, 2012). At the brain level, neuroimaging research that have found age-related neural differences in decision making during ultimatum games has primarily focused on identifying distinct regional brain activations, such as those in dorsolateral prefrontal cortex, anterior insula (e.g., Harlé & Sanfey, 2012; Gao et al., 2018), and ventromedial prefrontal cortex (e.g., Gu et al., 2015).

While prior neuroimaging research on the ultimatum game has successfully identified key brain regions involved in fairness-related decisions across age and social context, a focus on isolated regional activations may overlook the distributed nature of neural processing and the role of large-scale brain networks (Feng et al., 2021; S. M. Smith et al., 2009). Social decision making relies on coordinated interactions across systems, and mounting evidence suggests that aging alters both the internal organization of functional networks and the way these networks communicate with task-relevant brain regions (Andrews-Hanna et al., 2007; Schlesinger et al., 2017). To fully understand age-related differences in responses to social context, it is therefore necessary to examine how connectivity between large-scale brain networks and socially relevant regions changes across the lifespan. Here, we focus on two networks that have been repeatedly implicated in aging, social cognition, and decision making: the default mode network (DMN) and the executive control network (ECN). These networks have shown age-related differences in activation and connectivity during social decision making (Fareri et al., 2020, 2022) and have been linked to broader constructs such as value-based choice, norm enforcement, and vulnerability to exploitation (D. Smith et al., 2025; Spreng et al., 2017). By examining network connectivity as a function of age and social context, we aim to clarify how fairness decisions are supported across the lifespan and how older adults may preserve normative behavior through adaptive neural strategies.

In the present study, we attempt to clarify and extend previous mixed behavioral results and contribute to the literature of age and social context-related differences in brain activation and network connectivity during ultimatum games. First, our social context conditions go beyond the widely used human versus computer partner paradigm (e.g., Batten et al., 2024; Harlé & Sanfey, 2012) by including two human partner conditions to manipulate partner similarity, which has been shown to relate to judgments of interpersonal trust and offer rejection rates (Alarcon et al., 2016; Bailey et al., 2013). At the behavioral level, we hypothesized that when interacting with human partners, younger adults will show increased tendency to accept relatively fair offers when interacting with age-similar vs. age-dissimilar partners; older adults’ tendency to accept relatively fair offers (i.e., smaller offer size measured in US dollars) will not differ according to partner age similarity (BH).

At the neural level, we examined both regional activation and network connectivity during social decision making. Building on prior empirical and meta-analytic work (e.g., Fehr & Camerer, 2007; Gabay et al., 2014), we investigated whether neural responses to unfair offers differed across age groups and partner types. We hypothesized that neural activation in distinct regions will be diminished in older adults during receipt of unfair offers from age-similar vs. age-dissimilar partners (NH1). We also hypothesized that age-dependent changes in neural responses to offers will be tied to differential changes in connectivity of both DMN and ECN with distinct brain regions (NH2). Furthermore, beyond investigating brain responses to the fairness of offers (i.e., offer size), we also explored whether patterns of brain activity are related to an individual’s behavioral sensitivity to fairness norm violations as measured by the individual random slopes from a multilevel logistic regression model predicting offer acceptance from offer size (see Methods section for detail) (NH3).

## Methods

### Participants and Procedure

Participants were recruited according to their age to represent our two groups of interest, younger adults aged 18–35 and older adults aged 65–80. Our sample size was planned *a priori* to collect at least 20 usable participants in each group for a total of at least 40 participants; this planned sample size was limited by available funding for data collection. Young adult participants were recruited primarily through the Temple University Psychology Department participant pool, while older adult participants were recruited locally in Philadelphia, PA via flyering and newspaper advertisements, and reaching out to community and senior centers. All participants were screened before data collection to rule out major psychiatric or neurologic illness as well as MRI contraindications. Older adults were screened to rule out dementia using the Telephone Interview for Cognitive Status (Brandt et al., 1988). Participants were further excluded if their average framewise displacement in their brain images exceeded the upper bound defined by the boxplot method (1.5 × Interquantile Range above the third quartile). This sampling procedure resulted in a final sample of 47 participants of 25 young adults (mean age = 23.40 years, SD = 4.01; 16 female, 9 male) and 22 older adults (mean age = 69.32 years, SD = 4.36; 11 female, 11 male). Participants recruited through the participant pool were compensated with course credit, while those from the community were compensated $25 per hour of participation with Amazon and/or Visa gift cards; to increase the ecological validity of the task, we additionally provided bonus payments based on one randomly chosen trial from the experimental session. This study was approved by the Temple University Institutional Review Board and all participants gave informed consent.

After assessing eligibility based on initial screening procedures, participants were invited to participate in our study. We collected various measures as part of a larger neuroimaging experiment (see Smith et al., 2024 for more details), but here we will report only the pertinent details for the ultimatum game. Participants completed two appointments. During the first appointment, which lasted approximately 90 minutes, they underwent a mock MRI scan to acclimate them to the scanner and help control for motion. They then completed a brief neuropsychological test battery, with older adults additionally completing a self-report measure of everyday functioning. At the beginning of the second appointment, participants played a practice version of the ultimatum game to reduce the number of trials missed during the scan. The remaining duration of this appointment was spent playing economic games in the scanner and completing post-scan tasks (not reported here), after which participants were thanked, compensated, and dismissed.

### Experimental Design

**Figure 1.**
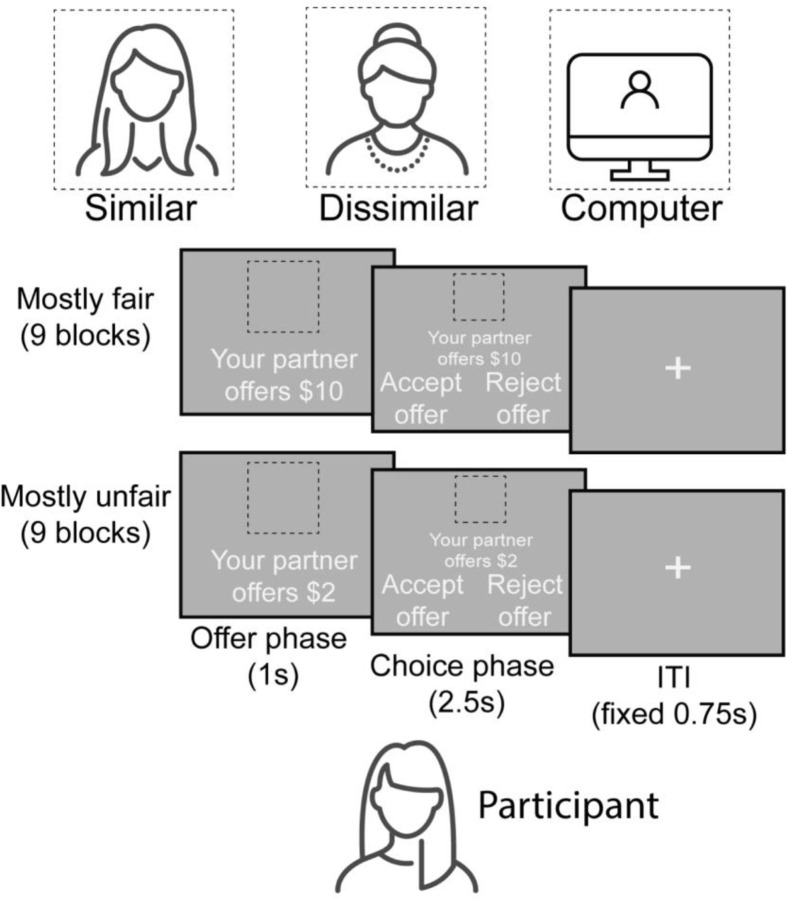
Depiction of the ultimatum game task that participants completed while undergoing neuroimaging. Participants received 144 total offers (18 blocks with 8 trials each) from three partners with whom they were told they would be splitting a $20 endowment: a computer (non-social control beyond the scope of the current investigation and excluded from analyses), a gender- and race-matched younger adult, and a gender- and race-matched older adult. Whether the human partner was considered a ‘similar’ or ‘dissimilar’ condition was therefore dependent upon the age of the participant. Each offer was either mostly fair (between 35% – 50% of the endowment value) or unfair (between 5% – 20%). Condition types of offer size and partner similarity were fully crossed, such that participants saw all offer sizes from all three partner types, though the order of the pairing of these conditions was randomized.

Participants played as responders in a multi-trial version of an ultimatum game wherein we manipulated both the fairness of proposed offers and the social context in which they were presented. Participants received 144 total offers (18 blocks with 8 trials each) from three partners with whom they were told they would be splitting a $20 endowment: a computer (which served as our non-social control), a gender- and race-matched younger adult, and a gender- and race-matched older adult. Whether the human partner was considered a ‘similar’ or ‘dissimilar’ condition was therefore dependent upon the age of the participant. On each trial, participants saw a screen with the offer value and the partner proposing that split as cued by a cartoon image of a younger adult, older adult, or computer (non-social control condition beyond the scope of the current investigation and excluded from analyses). Each offer was either mostly fair (i.e., between 35% – 50% of the endowment value) or unfair (between 5% – 20%). Following an interval of one second, participants had 2.5 seconds to either reject or accept the offer. See Figure 1 for a depiction of the task.

### Neuroimaging Data Collection and Preprocessing

Neuroimaging data were collected as part of a larger pilot study involving age-related differences in social and economic decision -making. Functional data were collected with a spatial resolution of 2.97×2.97×2.80 mm with a 2.02 s repetition time. All imaging data were first converted to BIDS format using HueDiConv (Halchenko et al., 2024) before further processing. Preprocessing of neuroimaging data was performed using fMRIPrep 20.2.3 (RRID:SCR_016216; (Esteban et al., 2019), which is based on Nipype 1.6.1 (RRID:SCR_002502; (Gorgolewski et al., 2011). Details of fMRI image acquisition, fMRIprep processing, and the full dataset have been reported in our protocol paper and are openly available on OpenNeuro (D. V. Smith et al., 2024).

### Behavioral Analyses

To test our behavioral hypothesis (BH), we employed a hierarchical model comparison approach using a series of multilevel logistic regression analyses on the trial-by-trial choice data (accept or reject) (Table 1). This method was selected to account for the nested data structure and to systematically identify the most parsimonious model.

Our analyses began with a full model predicting the likelihood of offer acceptance. This initial model included fixed effects for offer size, partner similarity, participant age group (younger vs. older), all two-way interactions, and the critical three-way interaction among these variables (Model 1 in Table 1). If the three-way interaction was not significant, we proceeded by testing a more simplified model that included only the two-way interactions and the main effects (Models 2 & 3). If the two-way interactions were also non-significant, we would then examine a final model containing only the main effects of offer size (Model 4), partner similarity (Model 5), and age group (Model 6) after controlling for offer size. For all models, we included random intercepts and random slopes of offer size for each participant to account for individual baseline differences in decision -making. All behavioral analyses were conducted in R version 4.3.2 with the lme4 package (Bates et al., 2015).

### Quantifying Behavioral Sensitivity to Fairness Norm Violation

To investigate how brain activity relates to individual differences in behavioral sensitivity to fairness norm violations (NH3), we first quantified this behavioral tendency for each participant. We derived an individual-level metric from a series of multilevel logistic regression models. For each partner-similarity condition (e.g., similar-age partner, dissimilar-age partner), we fit a multilevel logistic regression model predicting the association between choosing to accept and fairness (i.e., offer size). We included both fixed and random intercept and slope of offer size in the multilevel models. From each model, we extracted an individual-specific slope for every participant, which was calculated as the sum of the fixed-effect slope of the offer size and the participant’s unique random-effect slope for offer size. This value represents that individual’s estimated sensitivity to fairness within that specific social context. To create a single metric capturing the influence of partner similarity, we calculated a difference score by subtracting the sensitivity value derived from the dissimilar partner condition from the sensitivity value derived from the similar partner condition. This final score represents each participant’s partner similarity-related sensitivity to fairness norm violation. This individual sensitivity score and its interaction with participant age were then included as additional covariates in our group-level whole-brain analyses (see Group-Level Whole-Brain Analyses subsection below) to identify neural correlates of this fairness norm related behavioral tendency. All brain-behavior analyses were conducted in R version 4.3.2 with the lme4 package (Bates et al., 2015).

### Neuroimaging Analyses

#### fMRI First-Level Modeling

Functional data were analyzed in FSL using a general linear model (GLM) with local autocorrelation correction (Woolrich et al., 2001). We first focused on task-related activation using a GLM consisting of nine regressors. We used three regressors of partner similarity (human similar, human dissimilar, computer) to model trials (duration = 3.5 s) within each of the partner blocks. We also included parametric modulators for each of these three regressors, where each trial within a block was weighted by the offer size (i.e., dollar value of the offer, which is proportional to fairness given a fixed endowment of $20) on that trial. Given that response time may vary across partner similarity conditions and participants, we included additional regressors modeling the response as a constant term and with a parametric modulator for the response time on each trial. Finally, we included a regressor to account for missed trials. Each of these regressors were convolved with the canonical hemodynamic response function.

Task-dependent changes in connectivity were examined using psychophysiological interaction (PPI) analysis (Friston et al., 1997; D. V. Smith et al., 2016). Regions exhibiting significant PPI effect can be interpreted as showing a context-specific modulation of effective connectivity, though we note that the directionality of such an effect is ambiguous without additional analyses (Friston, 2011). Given our goal of examining DMN and ECN connectivity with other brain regions, we elected to use a network PPI approach that is not limited to single seed regions (Dobryakova & Smith, 2022; Utevsky et al., 2017). We conducted two separate models using the 10 networks characterized in prior work (S. M. Smith et al., 2004): one with the ECN as the primary network of interest (with the remaining nine networks, including DMN, as covariates) and one with DMN as the primary network of interest (with the remaining 9 networks, including ECN, as covariates). Each network time course was extracted using the spatial regression component of the dual regression approach and then added to the task-related activation model described above. We formed the PPI regressors by multiplying each of the 9 task regressors by the primary network regressor, which produced a total of 28 regressors for each model.

In both activation and connectivity models, we included a common set of confound regressors derived from the output of fMRIprep. Specifically, we included six regressors for head motion parameters (rotations and translations), the first six aCompCor components explaining the most variance, non-steady state volumes, and the framewise displacement across time. We also included a set of discrete cosine basis functions to apply a high-pass filter (128 s cut-off).

#### Group-Level Whole-Brain Analyses

To identify age-related neural responses to offer size, partner similarity, and sensitivity to fairness norm violation, we conducted group-level whole-brain analyses of activation and network connectivity. Group-level whole-brain analyses were carried out using FLAME (FMRIB’s Local Analysis of Mixed Effects) Stage 1 and Stage 2 (Woolrich et al., 2004). We implemented two types of group-level models focused on the contrast between similar and dissimilar partners (similar partner > dissimilar partner) as the dependent variable. The first model examined the interaction between age and partner similarity (NH1 & 2) and included regressors for age group (younger and older adults) and control covariates of gender, temporal signal to noise ratio (tSNR), mean framewise displacement, and mean response time to control for potential confounding effects of participant demographics and data quality measures. The second model examined the interaction between age, partner similarity, and fairness norm violation sensitivity interactions (NH3) and included the same covariates as the first model plus two additional regressors for interaction between fairness norm violation sensitivity and participant age group (i.e., sensitivity of similar minus dissimilar partner x younger adults, sensitivity of similar minus dissimilar partner x older adults). All z-statistic images were thresholded and corrected for multiple comparisons using an initial cluster-forming threshold of Z > 3.1 followed by a whole-brain corrected cluster-extent threshold of p < 0.05, as determined by Gaussian Random Field Theory (Flandin & Friston, 2019).

## Results

### Offer Size and Offer Acceptance

**Figure 2.**
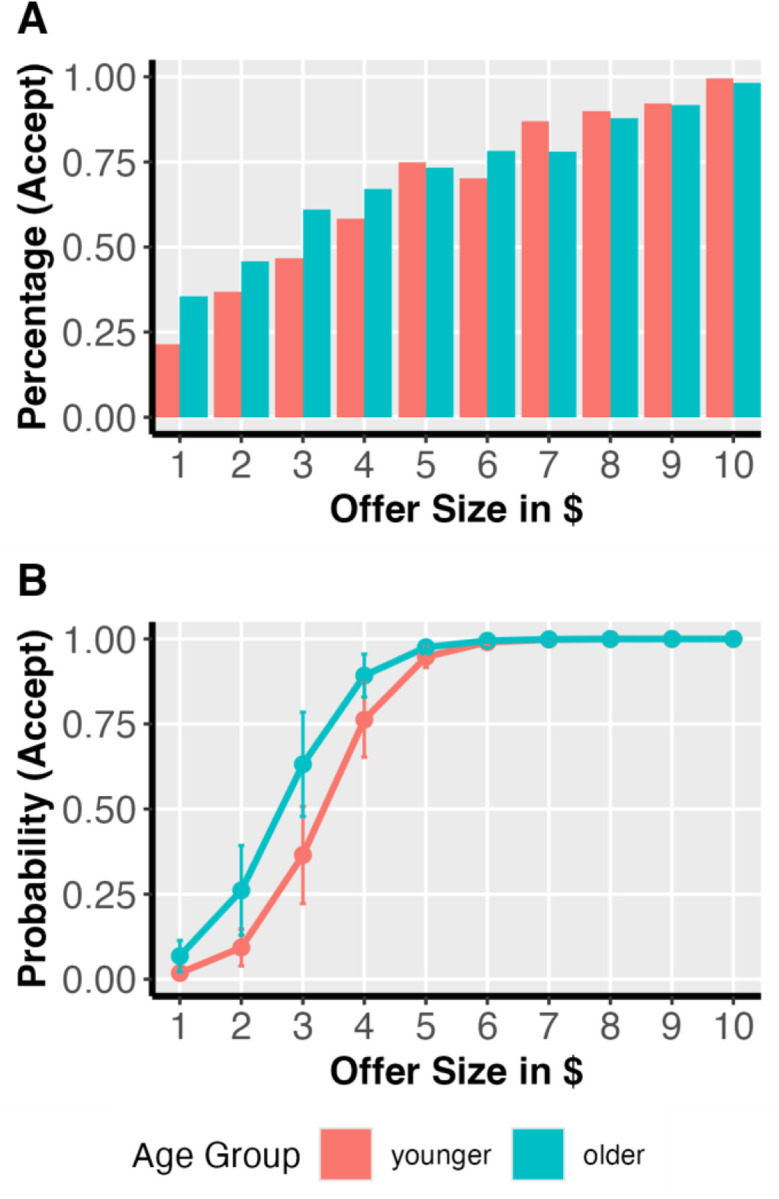
Positive relationship between offer fairness and acceptance probability. The figure displays the probability of participants accepting across offer size. The x-axis represents the size of the offer, indicating its level of fairness, while the y-axis shows the percentage of acceptance (Panel A) and mean probability of acceptance (Panel B). Error bars represent the standard error of the mean for each offer size. A multilevel logistic regression analysis revealed a significant main effect of offer size (beta = 1.65, *p* < .001).

For our primary analyses, we first examined if choice decisions are correlated with fairness, participant age group, and game partner’s similarity to the participant. We found a significant main effect of fairness (Figure 2). Participants are more likely to accept an offer as the fairness increases (beta = 1.65, p < 0.001). We did not find any age group and partner similarity moderation on the positive correlation between offer size and offer acceptance (offer size * age group * partner similarity, model did not converge; offer size * age group, beta = −.14, p = .637; offer size * partner similarity, beta = −.06, p = .227. Table 1) (BH).

### Age and Partner-Related Neural Response to Fairness

**Figure 3.**
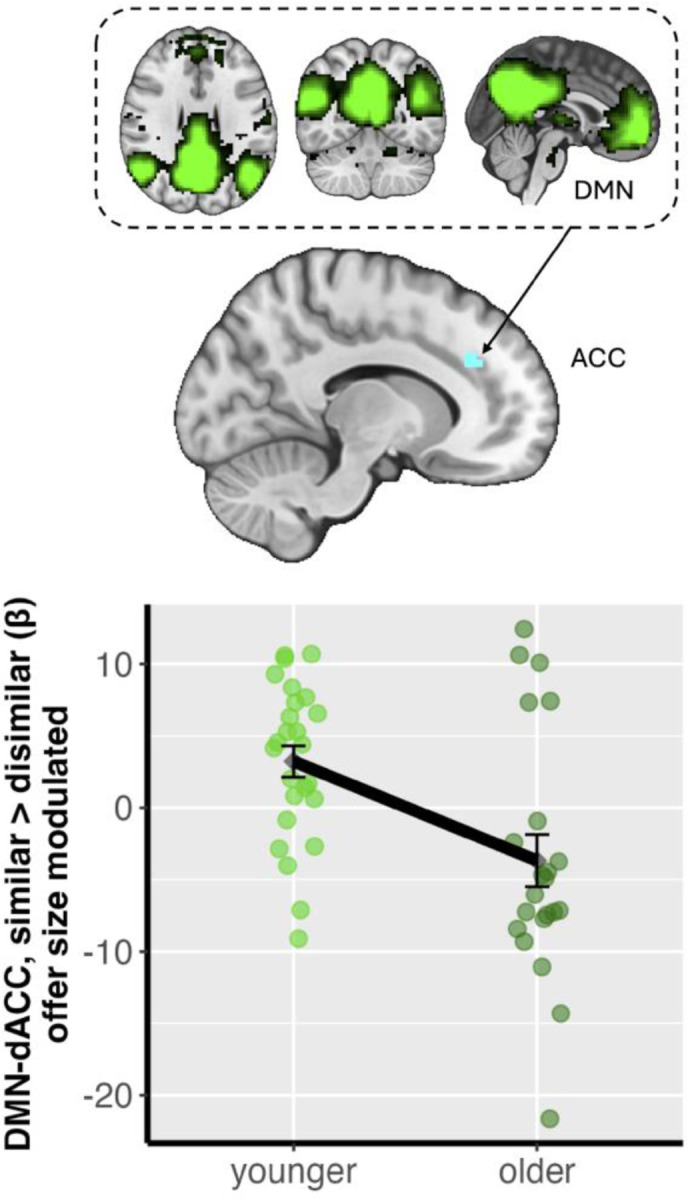
Age group and partner similarity effects on offer size modulated Default Mode Network (DMN)-anterior cingulate cortex (ACC) connectivity. The x-axis represents the two Age Groups (25 Younger Adults: 18–35 years; 22 Older Adults: 65–80 years). The y-axis displays the individual-level parameter estimates (beta) reflecting offer size-modulated DMN-ACC connectivity difference between the ‘similar partner’ and ‘dissimilar partner’ conditions (similar > dissimilar, ACC MNIxyz = −13.3, 34, 27.8; cluster = 26 voxels, p = .023). Each dot represents a single participant’s mean connectivity value for this contrast. Error bars indicate the standard error of the mean for each age group. A positive value on the y-axis signifies stronger offer size-modulated DMN-ACC connectivity when interacting with a similar partner compared to a dissimilar partner. This figure visually demonstrates opposing patterns of social context-dependent DMN-ACC connectivity between younger and older adults. Brain image is thresholded and corrected for multiple comparisons using an initial cluster-forming threshold of Z > 3.1 followed by a whole-brain corrected cluster-extent threshold of p < 0.05.

One focus of our study is how fairness-related brain activations may differ across age groups and game partner similarity. To answer this question, we conducted whole-brain activation (NH1) and DMN and ECN network connectivity analyses (NH2). For whole-brain activations (NH1), we did not find any significant interaction between partner similarity and participant age group. For brain network connectivity analyses (NH2), we found a significant interaction between participant age group and partner similarity (contrast: [young_similar-young_dissimilar] > [old_similar-old dissimilar]) in fairness-related DMN-anterior cingulate gyrus (ACC) connectivity (Figure 3). Specifically, younger adults exhibited a stronger fairness-modulated DMN-ACC connectivity when interacting with a similar versus dissimilar partner, whereas older adults demonstrated the opposite pattern (i.e., stronger DMN-ACC connectivity when interacting with a dissimilar versus similar partner; contrast = [young_similar-young_dissimilar] > [old_similar-old dissimilar], MNIxyz = −13.3, 34, 27.8; cluster = 26 voxels, p = .023, corrected). We did not find any significant interaction between participant age and partner similarity in ECN network connectivity.

### Neural Response and Behavioral Sensitivity to Fairness Norm Violation

**Figure 4.**
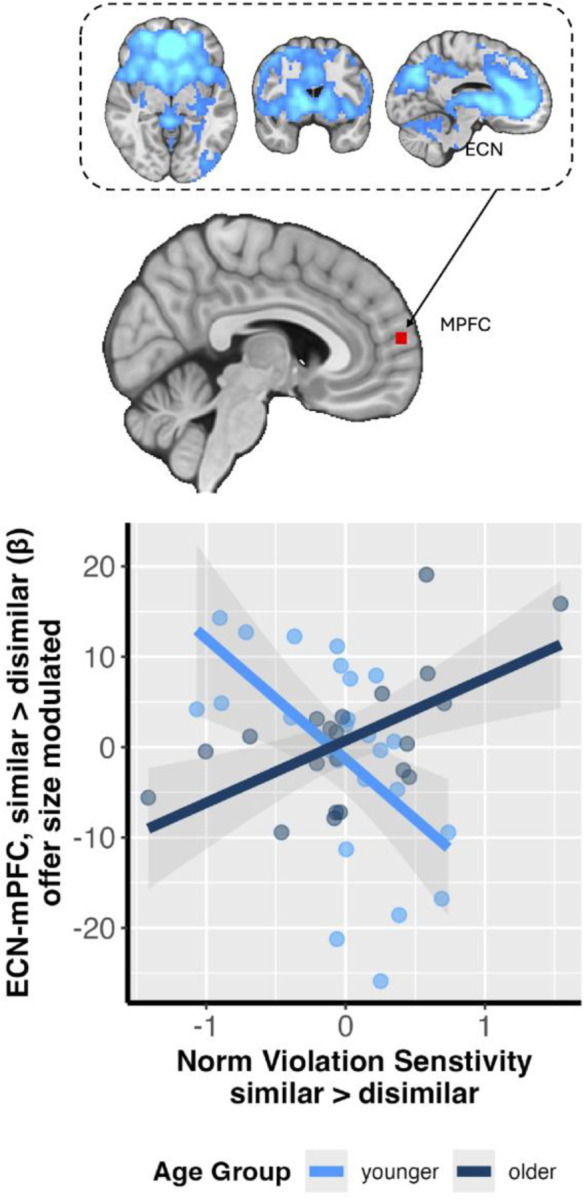
Brain-behavior relationship between ECN-mPFC connectivity and fairness norm violation sensitivity. The x-axis represents individual participant’s norm violation sensitivity difference between similar and dissimilar partners. The y-axis displays the individual-level parameter estimates (beta) reflecting offer size-modulated ECN-mPFC connectivity difference between the ‘similar partner’ and ‘dissimilar partner’ conditions (similar > dissimilar, mPFC MNIxyz =4.58, 60.7, 11.7; cluster = 23 voxels, p = .029). Light blue dots represent individual younger adult participants, while dark blue dots represent individual older adult participants. The solid lines indicate the linear fitted regression lines for each age group. The shaded regions around each fitted line denote the estimated standard error of the fitted line. This figure visualizes the opposing correlations between behavioral fairness sensitivity and neural differentiation in ECN-mPFC connectivity between similar and dissimilar partners across younger and older adults. MPFC image is thresholded and corrected for multiple comparisons using an initial cluster-forming threshold of Z > 3.1 followed by a whole-brain corrected cluster-extent threshold of p < 0.05.

In addition to the interaction between age group and partner similarity, we were also interested in whether behavior-level individual differences in sensitivity to fairness norm violation were associated with brain activation beyond fairness, age group, and partner similarity (NH3). To test this, we first estimated each participant’s sensitivity to fairness norm violation using participant-specific slopes from a multilevel logistic regression model (i.e., the sum of group-level fixed offer size effect and a participant’s unique corresponding random slope). A steeper positive slope indicates a greater sensitivity to the violation of a participant’s fairness norm. We then introduced these participant-specific fairness sensitivity slopes as a covariate into the group-level models for DMN and ECN connectivity analyses. We found a significant violation sensitivity effect in offer size-modulated ECN-mPFC connectivity related to age group and partner similarity ([older_similar-older_dissimilar] > [young_similar-young_dissimilar]) (Figure 4), such that younger adults’ behavioral sensitivity to fairness was negatively associated with ECN-mPFC connectivity difference between similar and dissimilar partners, whereas older adults showed a positive correlation (contrast = [young_similar-young_dissimilar] > [old_similar-old dissimilar], MNIxyz =4.58, 60.7, 11.7; cluster = 23 voxels, p = .029, corrected). That is, for younger adults, those who were more sensitive to fairness norms showed reduced ECN-mPFC connectivity when interacting with similar partners compared to dissimilar partners. In contrast, older adults demonstrated a reversed brain-behavior relationship, where those more sensitive to fairness exhibited heightened ECN-mPFC connectivity with similar partners relative to dissimilar partners.

## Discussion

The present study investigated how age and social context of partner identity modulate the neurobehavioral correlates of fairness-related economic decision -making. Although younger and older adults did not differ in their choice behavior as they consistently preferred fairer offers regardless of partner similarity (BH), their underlying neural mechanisms diverged (NH2 & 3). Specifically, younger adults showed increased DMN-anterior cingulate connectivity compared to older adults when considering offers from a similar partner (NH2). We also found that context-dependent connectivity between the ECN and mPFC was associated with behavioral sensitivity to fairness. However, this association depended on age, where older adults exhibited positive association, the opposite of younger adults’ negative association between ECN-mPFC and behavioral sensitivity to fairness (NH3). Collectively, these dissociations between behavioral and neural response to social context across age suggest that younger and older adults employ different neurocognitive strategies to navigate economic decisions across social context; older adults may engage distinct compensatory or reprioritized neural systems to achieve outcomes equivalent to those of younger adults.

Our neuroimaging results provide insights into the functional reorganization of brain networks among older adults in relation to social economic decision making. A primary finding was the age-related reversal in DMN-ACC connectivity associated with partner similarity, where younger adults exhibited heightened connectivity when considering offers from a similar partner, while older adults showed the inverse pattern. While intrinsic DMN connectivity has been indicated to often decline with age (Ng et al., 2016), our findings reveal a task-based functional reconfiguration that is critically sensitive to social context. This pattern is consistent with theories like the Default-Executive Coupling Hypothesis of Aging, which posits that older adults may leverage reduced network segregation as an adaptive strategy to support goal-directed cognition (Turner & Spreng, 2015). This neural recalibration was further illuminated by our second key finding: an interaction involving ECN-mPFC connectivity, individual sensitivity to fairness norm violation, age, and the social context of partner similarity. The mPFC is a critical hub for socio-affective processing (Lieberman et al., 2019; Tovar & Chavez, 2021), and its functional community structure is known to become less specialized and more interconnected with other networks during decision -making in older adults (Moussa et al., 2014; Ranjbar-Slamloo et al., 2025). Our results provide evidence for functional consequences for this known age-related reorganization: altered ECN-mPFC connectivity among older adults is meaningfully tied to sensitivity of individual fairness norms, but in a direction opposite to that of younger adults. Taken together, these findings cohesively implicate age-related neural adaptation, suggesting that the mechanisms supporting fairness-related decision making are not merely degraded by age, but are fundamentally recalibrated in relation to both external social context and stable internal dispositions. Importantly, those older adults whose neural recalibration is less effective or misaligned with the demands of external social contexts and internal dispositions may become more vulnerable to scams that exploit social cues and manipulate individual biases (e.g., Spreng et al., 2017). The current study did not include direct investigation of the association between age-related brain responses to social context and fraud victimization or risk for fraud. Future research incorporating such fraud measures would help to clarify the real-world implications of age and context-dependent neural responses during social decision making.

Notably, the age and social context-dependent patterns of neural activity we found stood in stark contrast to our behavioral findings, which showed no significant differences in choice behavior between age groups or partner identities. This dissociation between behavioral outcomes and their underlying neural substrates is a well-documented characteristic of cognitive aging and is broadly consistent with theories of neural compensation and functional reorganization (Morcom & Johnson, 2015). Previous research has demonstrated that older adults can achieve task performance comparable to or even better than their younger counterparts by recruiting different or additional neural resources (e.g., Bagarinao et al., 2019; Knights et al., 2024). The age-dependent opposing patterns of DMN-ACC and ECN-mPFC connectivity that we observed may represent such a compensatory shift. While younger adults appear to engage these networks in a manner sensitive to partner similarity, older adults may successfully regulate their behavior to align with social context and fairness norms as younger adults do by engaging these same networks in a different manner. This suggests that older adults can preserve behaviors similar to those of younger adults by adapting their neurocognitive strategies to overcome age-related changes in the baseline function and connectivity of key brain networks like the DMN and ECN.

While our findings illustrate a brain-behavior dissociation wherein older adults recruit distinct neural strategies to achieve normative social decision making, several limitations should be noted. First, we acknowledge that the sample size is relatively modest, which may affect the generalizability of our results and the statistical power to detect more subtle effects (Baranger et al., 2023). Second, our cross-sectional design precludes inferences about longitudinal aging trajectories, as the observed age differences could reflect cohort effects rather than the aging process per se (Argiris et al., 2021). Third, our design did not include a subjective measure to validate the social context manipulation of partner similarity. We therefore cannot definitively confirm that participants perceived age-similar partners as similar and more socially close than their age-dissimilar counterparts. Finally, we note that the observed interaction between participant age, partner similarity, and behavioral sensitivity to fairness may alternatively reflect an interaction between partner age and fairness sensitivity. That is, the neural patterns we attribute to age-related differences in decision making could instead—or additionally—be driven by characteristics of the proposing partner, such as their age. Future work could adjudicate between these interpretations by systematically measuring and modeling other dimensions of perceived similarity between participants and their partners (e.g., social closeness, gender, shared values, or group membership).

## Conclusion

In summary, the present study advances our understanding of how aging shapes the neural mechanisms underlying fairness-related social decision -making. While behavioral choices remained stable across age groups, our findings reveal a reorganization of functional brain networks, particularly involving the DMN, ACC, ECN, and mPFC, which adaptively support fairness processing in distinct social contexts. This neural flexibility may reflect compensatory or strategic shifts that enable older adults to maintain normative social behavior despite age-related changes in brain function. Future research should further elucidate the causal dynamics of these network adaptations and explore their generalizability across diverse social and cultural settings. By integrating behavioral, neural, and individual difference measures, this work lays important groundwork for a more nuanced model of socio-cognitive aging that bridges brain function with real-world age-related maladaptive social behavior such as fraud risk and victimization.

## Figures and Tables

**Table 1.** Models and Estimates (fixed effects, *p<0.001)

Models (factors included)	Model 1 (offer, age, similarity)	Model 2 (offer, age)	Model 3 (offer, similarity)	Model 4 (offer, age as control)	Model 5 (offer, similarity as control)	Model 6 (offer)
Regressors	Beta Estimates (Standard Error)					
offer * age * similarity	Failed to converge					
offer * age		−0.144 (0.305)				
offer * similarity			−0.057 (0.047)			
offer		1.721* (0.218)	1.686* (0.172)	1.660* (0.17)	1.655* (0.17)	1.655* (0.170)
age		1.523 (2.133)		0.969 (1.783)		
similarity			0.271 (0.239)		0.022 (0.123)	
intercept		−5.717* (1.476)	−5.152* (1.092)	−5.463* (1.364)	−5.024* (1.089)	−5.013* (1.087)
**Observations**		4439.00	4439.00	4439.00	4439.00	4439.00
**Log Likelihood**		−943.05	−942.59	−943.15	−943.28	−943.30
**AIC**		1900.11	1899.19	1898.31	1898.57	1896.60
**BIC**		1944.89	1943.98	1936.69	1936.96	1928.59

* p<0.001

similarity = partner similarity; offer = offer size; age = participant age
